# Comparative chloroplast genomics and phylogenetic analysis of *Oreomecon nudicaulis* (Papaveraceae)

**DOI:** 10.1186/s12863-024-01236-8

**Published:** 2024-05-30

**Authors:** Qingbin Zhan, Yalin Huang, Xiaoming Xue, Yunxia Chen

**Affiliations:** 1 College of Criminal Science and Technology, Nanjing Police University, Nanjing, China; 2 Key Laboratory of State Forestry and Grassland Administration on Wildlife Evidence Technology, Nanjing, China

**Keywords:** *O. nudicaulis*, Chloroplast genome, Structural characteristics, Systematic evolution

## Abstract

*Oreomecon nudicaulis*, commonly known as mountain poppy, is a significant perennial herb. In 2022, the species *O. nudicaulis*, which was previously classified under the genus *Papaver*, was reclassified within the genus *Oreomecon*. Nevertheless, the phylogenetic status and chloroplast genome within the genus *Oreomecon* have not yet been reported. This study elucidates the chloroplast genome sequence and structural features of *O. nudicaulis* and explores its evolutionary relationships within Papaveraceae. Using Illumina sequencing technology, the chloroplast genome of *O. nudicaulis* was sequenced, assembled, and annotated. The results indicate that the chloroplast genome of *O. nudicaulis* exhibits a typical circular quadripartite structure. The chloroplast genome is 153,903 bp in length, with a GC content of 38.87%, containing 84 protein-coding genes, 8 rRNA genes, 38 tRNA genes, and 2 pseudogenes. The genome encodes 25,815 codons, with leucine (Leu) being the most abundant codon, and the most frequently used codon is AUU. Additionally, 129 microsatellite markers were identified, with mononucleotide repeats being the most abundant (53.49%). Our phylogenetic analysis revealed that *O. nudicaulis* has a relatively close relationship with the genus *Meconopsis* within the Papaveraceae family. The phylogenetic analysis supported the taxonomic status of *O. nudicaulis*, as it did not form a clade with other *Papaver* species, consistent with the revised taxonomy of Papaveraceae. This is the first report of a phylogenomic study of the complete chloroplast genome in the genus *Oreomecon*, which is a significant genus worldwide. This analysis of the *O. nudicaulis* chloroplast genome provides a theoretical basis for research on genetic diversity, molecular marker development, and species identification, enriching genetic information and supporting the evolutionary relationships among Papaveraceae.

**Supplementary Information:**

The online version contains supplementary material available at 10.1186/s12863-024-01236-8.

## Introduction

The Papaveraceae family, commonly known as the poppy family, is a diverse group of flowering plants primarily distributed in northern temperate regions, with a significant presence in the Mediterranean, Western Asia, Central Asia, East Asia, and southwestern regions of North America [1]. The majority of plants within the Papaveraceae famliy are annual or perennial herbs, and a few are shrubs or small trees. The leaves of these plants are typically alternate or opposite, lacking stipules, and often exhibit lobed edges. The flowers are bisexual, solitary, large and showy in shape, but have no fragrance. The entire plant is known for secreting white, yellow, or red latex [2, 3]. Globally, the Papaveraceae family is renowned for its remarkable diversity, encompassing approximately 42 genera and more than 700 species [4]. In China, the Papaveraceae family is widely distributed across various regions, with approximately 18 genera and 362 species, exhibiting the most extensive distribution in the southwestern part of the country [5].

The genus *Papaver* is a significant member of the Papaveraceae family, consisting of 70–100 species of cold-resistant annuals, biennials, and perennials native to temperate and cold regions across Eurasia, Africa, and North America [6–8]. This genus has been the subject of extensive research due to its economic, ornamental, and medicinal importance *Papaver* species are renowned for their vibrant flowers and have been cultivated for centuries as ornamental plants. Additionally, some *Papaver* species, such as *Papaver somniferum*, are of significant medicinal value due to their production of alkaloids, including morphine, codeine, and thebaine, which have potent analgesic and narcotic properties, making them essential in the pharmaceutical industry. *Papaver* sect. *Meconella* comprises 24 to 30 species distributed across the entire Arctic region, spanning from polar areas to mountain ranges [9].

In 2022, a new genus, *Oreomecon*, was established to address the prior classification of *Papaver* sect. *Meconella*, ensuring the monophyly of the genus *Papaver*. Subsequently, several species present in Europe, both native and alien, were transferred to this newly formed genus. Its inception aimed to address the prior classification of *Papaver sect. Meconella*, ensuring the monophyly of the genus *Papaver* [10]. Currently, the genus *Oreomecon* comprises six species: *Oreomecon alpina*, *O. anomala*, *O. crocea*, *O. miyabeana*, *O. nudicaulis*, and *O. radicata*. Among these species, *O. nudicaulis*, commonly known as Iceland poppy, mountain poppy, Icelandic corn poppy, orange-flowered poppy, and mountain tobacco, is a perennial herb characterized by its sturdy, unbranched rhizome and unique flowers. This wild poppy exhibits robust cold resistance and is frequently encountered in mountainous forest margins, grasslands, meadows, sand dune thickets, and ravines. Given its adaptability and ornamental value, *O. nudicaulis* is easily cultivated and holds substantial potential for use in gardens [11]. In addition to its ornamental value, the fruits and whole plants of *O. nudicaulis* are utilized in traditional Chinese medicine, particularly by the Mongolian ethnic group in China, for the treatment of persistent coughs, asthma, and chronic diarrhea [12].

Despite the reclassification of *O. nudicaulis* into the genus *Oreomecon*, there is limited information available on the phylogenetic status and chloroplast genome of this genus. Chloroplasts, which are significant organelles in plants responsible for photosynthesis, exhibit a notable degree of genome complexity. Chloroplast genomes have emerged as valuable tools for studying plant systematics, evolution, and phylogenetic relationships. These genomes not only encode enzymes and proteins necessary for photosynthesis but also harbor a substantial number of noncoding sequences, providing rich information for molecular biology and evolutionary studies. In particular, the conserved and nonrecombinant nature of the chloroplast genome makes it highly valuable for species identification, genetic relationship analysis and the study of biological evolution [12–16].

The genomic content of chloroplasts is rich with valuable information, making them ideal models for research, particularly in the fields of molecular markers, barcoding identification, plant phylogenetics, evolution, and comparative genomic studies [17–20]. The chloroplast genome is recognized for its greater conservation compared to nuclear or mitochondrial genomes in terms of genetic structure, gene content, and nucleotide sequence. Due to its highly conserved and nonrecombinant nature, the chloroplast genome serves as a valuable genetic resource for deducing evolutionary relationships at various taxonomic levels [21]. The typical circular chloroplast genome exhibits a conserved quadripartite structure comprising a large single-copy region (LSC) and a small single-copy region (SSC) separated by a pair of inverted repeats (IRs). Additionally, the majority of angiosperm chloroplast genomes are 110–170 kb in length [22].

The advent of high-throughput sequencing technologies, such as Illumina sequencing, has revolutionized the field of plant genomics. These technologies have enabled the rapid and cost-effective sequencing of complete chloroplast genomes, providing valuable insights into the evolutionary history and relationships among plant species. Comparative analyses of chloroplast genomes have been successfully used to resolve phylogenetic relationships at various taxonomic levels, from family to species. In the Papaveraceae family, several studies have investigated the chloroplast genomes of various genera, including *Papaver*, *Meconopsis*, and *Eschscholzia*. These studies have provided valuable insights into the evolutionary relationships and genome structure of these genera. However, to date, no study has reported the chloroplast genome of the genus *Oreomecon* or explored its phylogenetic position within the Papaveraceae family.

The present study utilized high-throughput sequencing technology to assemble and annotate the entire genome of *O. nudicaulis*, marking the first report of the chloroplast genome of this genus. Furthermore, this study explores the evolutionary relationships of *O. nudicaulis* within the Papaveraceae family through phylogenetic analysis. The specific objectives of this study are: (1) to assemble and annotate the complete chloroplast genome of *O. nudicaulis*; (2) to analyze the genome structure, gene content, and codon usage patterns of the *O. nudicaulis* chloroplast genome; (3) to identify microsatellite markers in the *O. nudicaulis* chloroplast genome; and (4) to investigate the phylogenetic position of *O. nudicaulis* within the Papaveraceae family using chloroplast genome data. Subsequent analyses encompassing genomic composition, structure, and phylogenetic relationships not only enhanced the genetic knowledge of the chloroplast genome within the Papaveraceae family but also established a foundation for the development of molecular markers, exploration of genetic diversity, and investigations into the origin and evolution of poppy plants.

## Materials and methods

### Plant material and DNA extraction

Fresh and well-grown *O. nudicaulis* plants used in this study were obtained from Shata, Yili, Xinjiang Uygur Autonomous Region, China. The samples were preserved in the plant laboratory of Nanjing Police University. The chloroplast genome data have been submitted to the NCBI website under accession number MW151698. Freshly fleshy stems of *O. nudicaulis* were collected, and the genomic DNA was extracted using the cetyltrimethylammonium bromide (CTAB) method. The purity and integrity of the DNA were assessed through 1% agarose gel electrophoresis [23]. DNA quantification was performed using a NanoDrop 2000 spectrophotometer (Thermo Fisher Scientific, USA) to ensure that the samples met the quality requirements for subsequent sequencing.

### Genome sequencing and assembly

Once the sample’s chloroplast genomic DNA passed quality control, library preparation was performed using the Illumina TruSeq DNA Sample Prep Kit (Illumina, USA) following the manufacturer’s instructions. The prepared library was then sequenced on the Illumina NovaSeq platform with paired-end reads of 150 base pairs. This sequencing process was carried out by Nanjing Jusen Huiyuan Biotechnology Co., Ltd. The raw sequencing data obtained from the Illumina platform were subjected to quality control using FastQC v0.11.9 (http://www.bioinformatics.babraham.ac.uk/projects/fastqc/). Low-quality reads and adapter sequences were removed using Trimmomatic v0.39. The chloroplast genome assembly was conducted using SPAdes v3.10.1 software (http://cab.spbu.ru/software/spades/).

### Genome annotation and analysis

The annotation of the *O. nudicaulis* chloroplast genome was performed using a combination of automated and manual methods. The initial annotation was carried out using the online tool GeSeq, which employs a BLAST-based approach to identify and annotate genes, tRNAs, and rRNAs. The coding sequences (CDSs) were predicted using Prodigal v2.6.3(https://www.github.com/hyattpd/Prodigal), while rRNA prediction was accomplished with HMMER v3.1b2 (http://www.HMMER.org/) [11], and tRNA prediction was conducted using ARAGORN v1.2.38 (http://130.235.244.92/ARAGORN/) [24]. The predicted genes and RNA structures were manually curated and verified using the BLAST tool against the NCBI nucleotide and protein databases. The boundaries of the inverted repeat (IR) regions were determined using the online tool IRscope. The circular chloroplast genome map was created using OGDRAW (https://chlorobox.mpimp-golm.mpg.de/OGDraw.html), which provides a user-friendly interface for visualizing and annotating organelle genomes.

### Codon usage and microsatellite analysis

Codon usage and relative synonymous codon usage (RSCU) in the *O. nudicaulis* chloroplast DNA were statistically analyzed using CodonW software and EMBOSS explorer (https://www.bioinformatics.nl/emboss-explorer/) [25]. Repeat sequences, including forward, palindrome, reverse, and complementary repeats, were detected using Vmatch v2.3.0 software (http://www.vmatch.de) with parameters set to a minimum length of 30 bp and a Hamming distance of 3. Simple sequence repeats (SSRs) were predicted using MISA v1.0 (http://pgrc.ipk-gatersleben.de/misa/misa.html) with the following parameters: mono-nucleotide repeat units with a minimum of 8, di-nucleotide repeat units with a minimum of 5, and tri-, tetra-, penta-, and hexa-nucleotide repeat units with a minimum of 3 [26].

### Chloroplast genome comparison and IR boundary analysis

Comparison analysis of the chloroplast genome of *O. nudicaulis* was conducted with three published species of the Papaveraceae family obtained from NCBI. The structural information of the chloroplast genome was documented using Microsoft Excel. Whole-genome comparisons were performed using mVISTA (https://genome.lbl.gov/vista/mvista/submit.shtml) [27]. Additionally, the IRscope visualization tool (https://irscope.shinyapps.io/irapp/) was used to analyze the boundary differences in the LSC, SSC, IRa, and IRb regions of the chloroplast genome between *O. nudicaulis* and its closely related species, including *Papaver somniferum*, *Papaver rhoeas*, and *Papaver orientale* [28].

### Phylogenetic analysis

To determine the phylogenetic position of *O. nudicaulis* within Ranunculales, 54 chloroplast genome sequences representing 7 genera of Papaveraceae and 8 genera of other families were downloaded from NCBI. To construct the species tree, we employed the OrthoFinder version 2.5.4, which is widely used for inferring phylogenetic relationships from orthologous genes. Alignments were performed using MAFFT software [29], and a maximum likelihood (ML) phylogenetic tree was constructed using FastTree software [30]. To calculate protein identity, we conducted a comparison between the protein sequences of each species and the homologous protein of *O. nudicaulis* using the NCBI blastp tool. Furthermore, we visualized the resulting phylogenetic tree, along with the protein identity heatmap, using the Evolview v3 platform (https://www.evolgenius.info/evolview/#/treeview). By incorporating the protein identity data into the phylogenetic tree visualization, the heatmap provides a visual representation of the protein sequence similarities among the species.

### Collinearity analysis

For collinearity analysis of the chloroplast genome of *O. nudicaulis* and related species, we compared the chloroplast genome sequences of 9 plants, namely, *Papaver orientale* (NC_037832), *Papaver pseudo-orientale* (NC_065210), *Papaver rhoeas* (NC_037831), *Papaver dubium* (NC_065205), *Papaver somniferum* (NC_029434), *Meconopsis integrifolia* (MK533647), *Meconopsis henrici* (MN488591), *Meconopsis horridula* (MK533646), and *Meconopsis racemosa* (MK533649). The custom Perl script and R packages genoPlotR and ComplexHeatmap were used to perform collinearity and phylogenetic analysis among *O. nudicaulis* and related species [31].

## Result

### Chloroplast genome structure analysis of *O. nudicaulis*

**Fig. 1:**
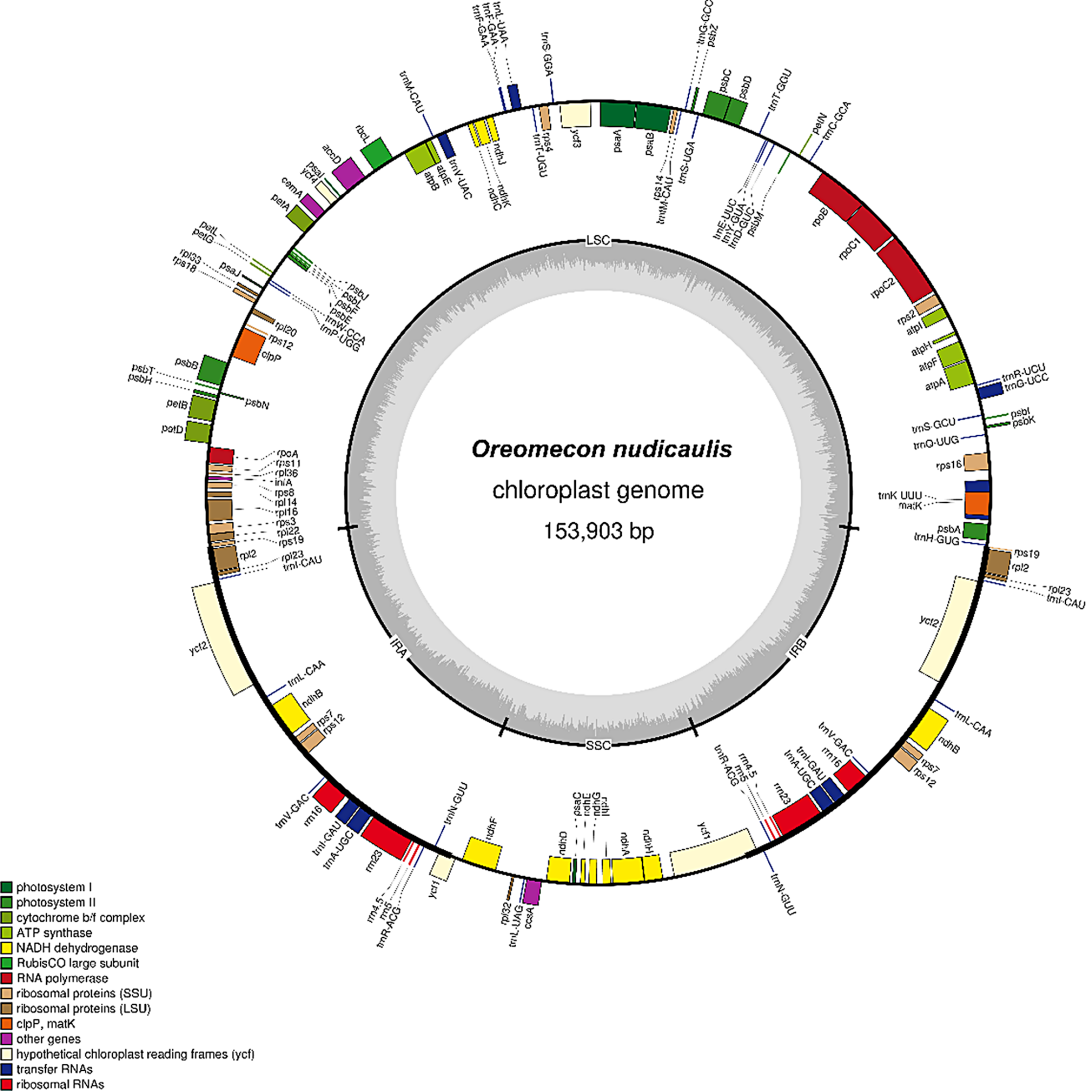
Gene map of the chloroplast genome of *O. nudicaulis*. The forward-coding genes are located beyond the perimeter of the circle, whereas the reverse-coding genes are located within its center. The inner gray circle symbolizes the GC content

The chloroplast genome of *O. nudicaulis* exhibits a typical circular quadripartite structure with a total length of 153,903 base pairs (bp) and a GC content of 38.87% (Fig. 1). Specifically, the large single-copy (LSC) region is 83,676 bp long with a GC content of 37.33%, the small single-copy (SSC) region is 18,867 bp long with a GC content of 33.87%, and the inverted repeat regions IRa and IRb are both 25,680 bp long with a GC content of 43.22%. The chloroplast genome of *O. nudicaulis* was annotated with a total of 132 genes, including 84 protein-coding genes, 8 rRNA genes, 38 tRNA genes, and 2 pseudogenes. Among them, 18 genes were present in double copies, and 18 genes contained introns. Notably, the *clpP* and *ycf3* genes have 2 introns, while the remaining 16 genes have 1 intron each. *ycf1* and *rps19* are pseudogenes (Table 1).

**Table 1:** Gene annotation of the chloroplast genome of *O. nudicaulis*

Category of genes	Group of genes	Name of genes
Photosynthesis	Subunits of photosystem I	*psaA, psaB, psaC, psaI, psaJ*
	Subunits of photosystem II	*psbA, psbB, psbC, psbD, psbE, psbF, psbH, psbI, psbJ, psbK, psbL, psbM, psbN, psbT, psbZ*
	Subunits of NADH dehydrogenase	ndhA*, ndhB*(2), ndhC, ndhD, ndhE, ndhF, ndhG, ndhH, ndhI, ndhJ, ndhK
	Subunits of cytochrome b/f complex	petA, petB*, petD*, petG, petL, petN
	Subunits of ATP synthase	atpA, atpB, atpE, atpF*, atpH, atpI
	Large subunit of rubisco	*rbcL*
Self-replication genes	Large subunit of ribosomal	rpl14, rpl16*, rpl2*(2), rpl20, rpl22, rpl23(2), rpl32, rpl33, rpl36
	Small subunit of ribosomal dependent RNA polymerase	#rps19, rps11, rps12**(2), rps14, rps16*, rps18, rps19, rps2, rps3, rps, rps7(2), rps8
	DNA dependent RNA polymerase	rpoA, rpoB, rpoC1*, rpoC2
	Ribosomal RNAs	*rrn16(2), rrn23(2), rrn4.5(2), rrn5(2)*
	Transfer RNAs	trnA-UGC*(2), trnC-GCA, trnD-GUC, trnE-UUC, trnF-GAA(2), trnG-GCC, trnG-UCC*, trnH-GUG, trnI-CAU(2), trnI-GAU*(2), trnK-UUU*, trnL-CAA(2), trnL-UAA*, trnL-UAG, trnM-CAU, trnN-GUU(2), trnP-UGG, trnQ-UUG, trnR-ACG(2), trnR-UCU, trnS-GCU, trnS-GGA, trnS-UGA, trnT-GGU, trnT-UGU, trnV-GAC(2), trnV-UAC*, trnW-CCA, trnY-GUA, trnfM-CAU
Other genes	Maturase	*matK*
	ATP ATP-dependent protease subunit P	clpP**
	Envelope membrane protein	*cemA*
	Subunit of Acetyl-CoA carboxylase	*accD*
	c-type cytochrome synthesis gene	*ccsA*
	Translation initiation factor	*infA*
Genes of unknown function	Conserved hypothetical chloroplast ORF	#ycf1, ycf1, ycf2(2), ycf3**, ycf4

*Note* “*”One intron; “**”Two introns; “(2)”Two copies; “#” Pseudogene

### Characteristics of protein-coding genes and codon usage in *O. nudicaulis*

**Fig. 2:**
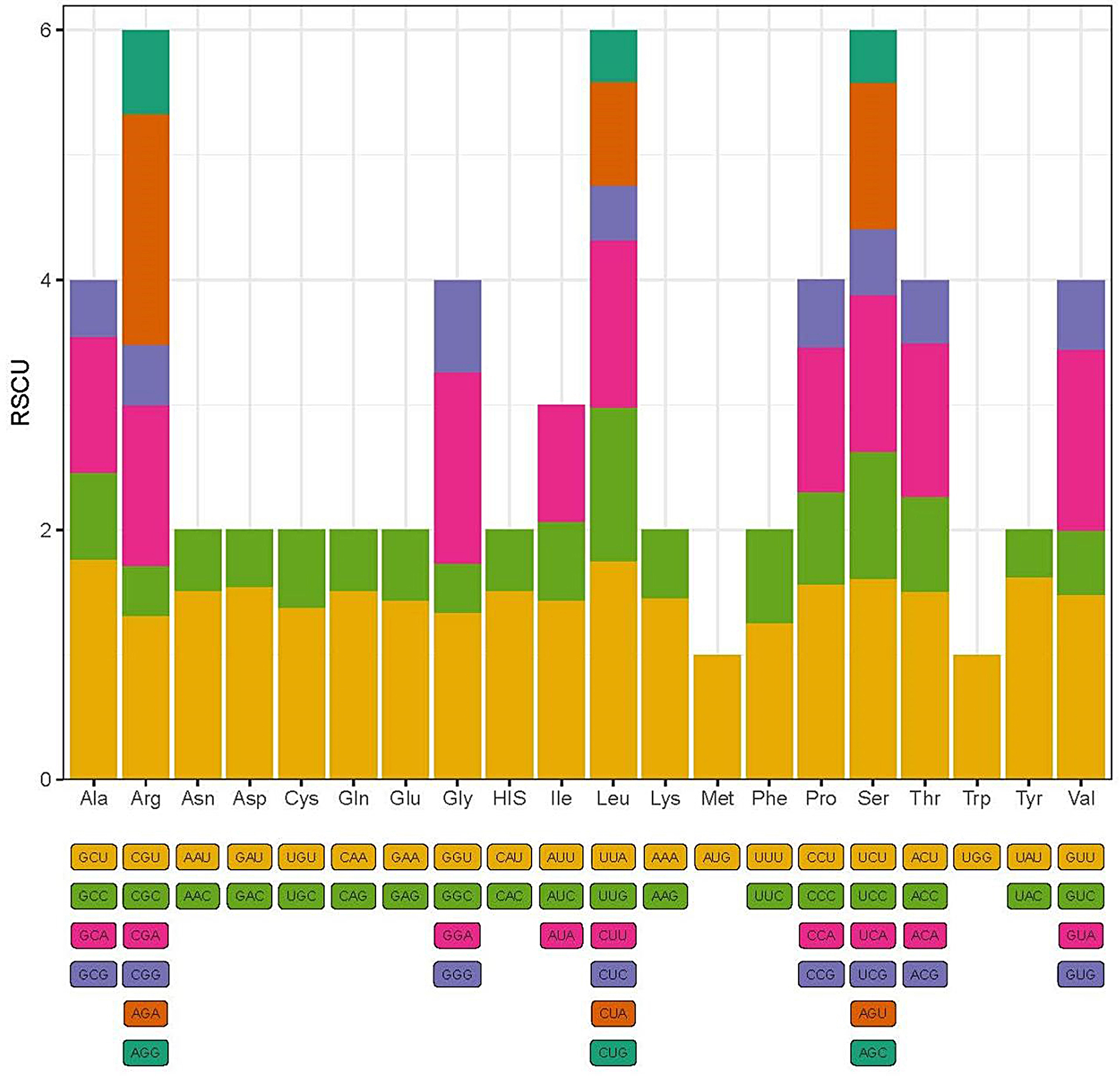
Frequency of amino acid use in protein-coding genes of *O. nudicaulis*. The boxes below represent the total RSCU values of all codons encoding each amino acid; the heights of the columns above them represent the total RSCU values of all codons

The chloroplast genome of *O. nudicaulis* encompasses a total of 65 codons encoding amino acids, amounting to 25,815 codons in total. Among these codons, those encoding leucine (Leu) are the most abundant, with 2,702 codons, constituting 10.47% of the total, while codons encoding cysteine (Cys), excluding stop codons, are the least frequent, with 305 codons, comprising only 1.18% of all codons. Within the 65 codons identified in *O. nudicaulis*, the codon usage for tryptophan (Trp: UGG) (Met: AUG) exhibited a value of 1, indicating unbiased usage. Among the codons with RSCU values greater than or less than 1, 32. The most frequently used codon was AUG (Fig. 2), encoding methionine (Met), while the least commonly used codon was GUG, also encoding methionine. Among the three stop codons, UAA had the highest usage rate, with an RSCU value of 1.5326.

### Analysis of repetitive sequences and SSRs in the *O. nudicaulis* chloroplast genome

**Fig. 3:**
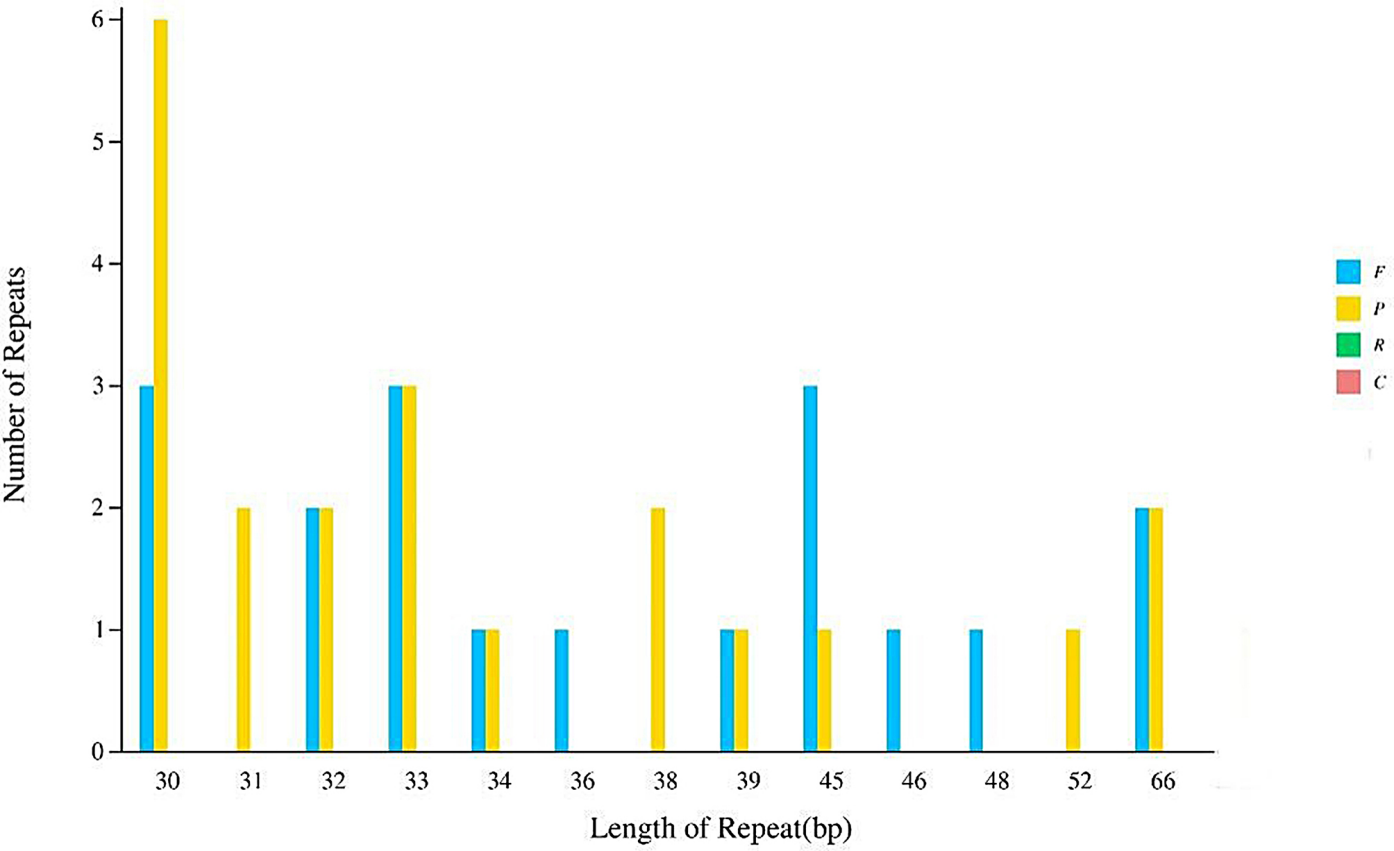
Distribution of Repeated Sequences in the Complete Chloroplast Genome of *O. nudicaulis*. This bar graph displays the length of various repeated sequences (in base pairs) identified within the chloroplast genome of *O. nudicaulis*. The x-axis represents the length of the repeat sequences, ranging from 30 to 25,680 base pairs. The y-axis denotes the frequency of each repeat length. The types of repeats are classified into Forward (F), Reverse (R), Complement (C), and Palindromic (P) sequences, as indicated by the respective annotations

The chloroplast genome of *O. nudicaulis* harbors a total of 39 scattered repetitive sequences with lengths exceeding 30 base pairs (bp). Among these, 18 were forward repeat sequences, and 21 were palindromic repeat sequences; no reverse repeat sequences or complementary repeat sequences were detected. The most abundant repetitive sequences were those with a length of 30 bp, totaling 9 (3 forward and 6 palindromic), constituting 23.07% of the total (Fig. 3).

**Fig. 4:**
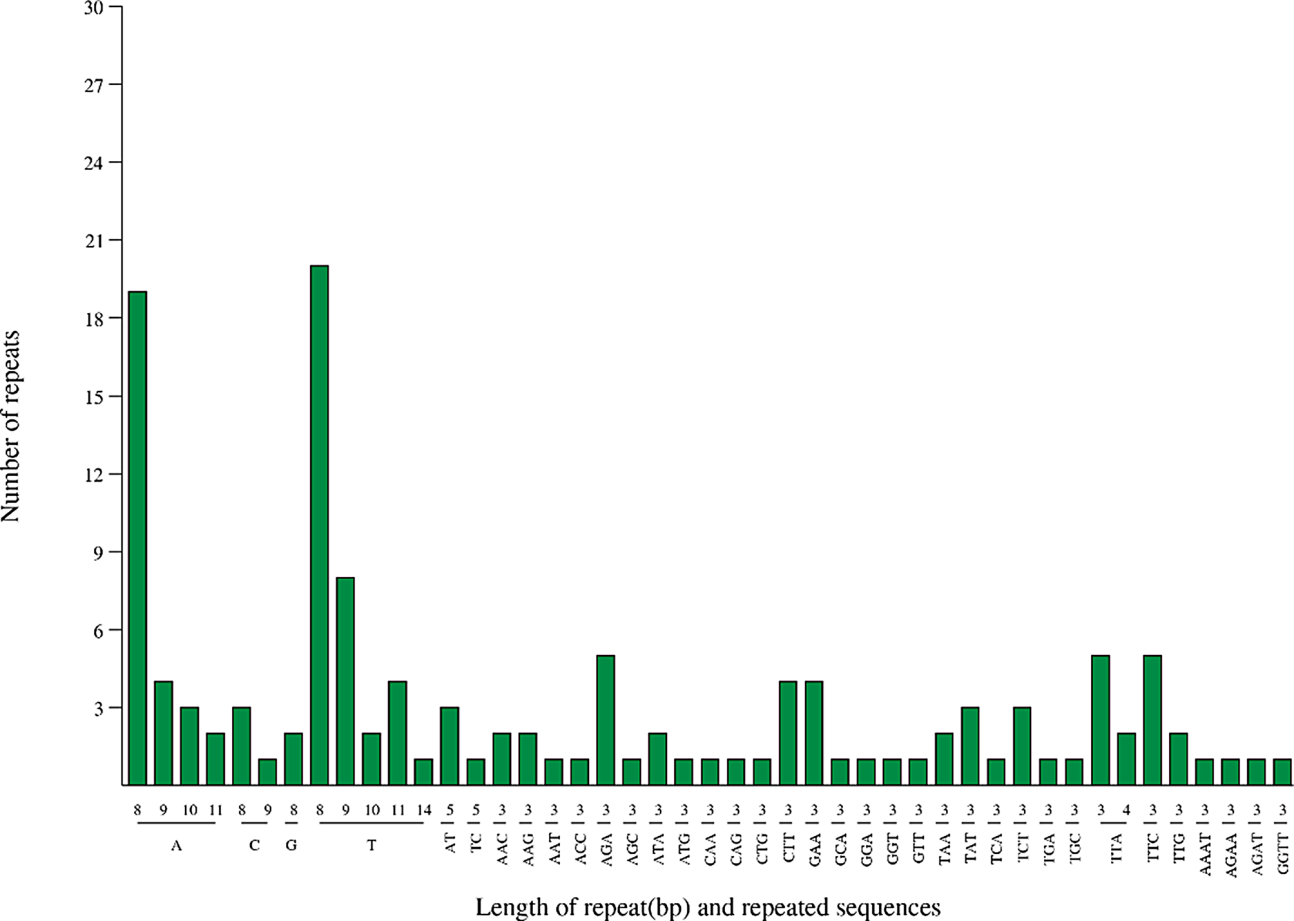
Numerical statistics of SSRs in the complete chloroplast genome of *O. nudicaulis.* This figure presents a bar graph illustrating the distribution of SSRs by length and the number of repeats. The x-axis represents the length of the repeat in base pairs (bp), while the y-axis indicates the number of SSRs found within each length category

The chloroplast genome of *O. nudicaulis* encompasses a total of 129 microsatellite sequences. Among these sequences, mononucleotide repeat sequences were the most abundant, comprising 69 sequences and accounting for 53.49%. There were 4 dinucleotide repeat sequences, 55 trinucleotide repeat sequences, and 4 tetranucleotide repeat sequences. No pentanucleotide, hexanucleotide, heptanucleotide, or octanucleotide repeat sequences were detected. Notably, these microsatellite sequences exhibited a pronounced bias toward the A/T base composition (Fig. 4).

An analysis of the distribution of SSRs in the chloroplast genome of *O. nudicaulis* revealed that 49.6% of SSRs were located in the large singlecopy (LSC) region, 22.5% in the small single-copy (SSC) region, and 27.9% in the inverted repeat (IR) region, indicating an uneven distribution. Additionally, there was variation in the distribution of SSRs among different functional gene regions, with 80 located in exonic regions, 6 in intronic regions, and 43 in intergenic regions (Table 2).

**Table 2:** Distribution of SSRs in the complete chloroplast genome of *O. nudicaulis*

Region	Number	Proportion(%)	Exon	Intron	Intergenic
LSC	64	49.6	28	6	30
SSC	29	22.5	22	0	7
IR	36	27.9	30	0	6

### Analysis of the IR boundaries in the *O. nudicaulis* chloroplast genome

**Fig. 5:**
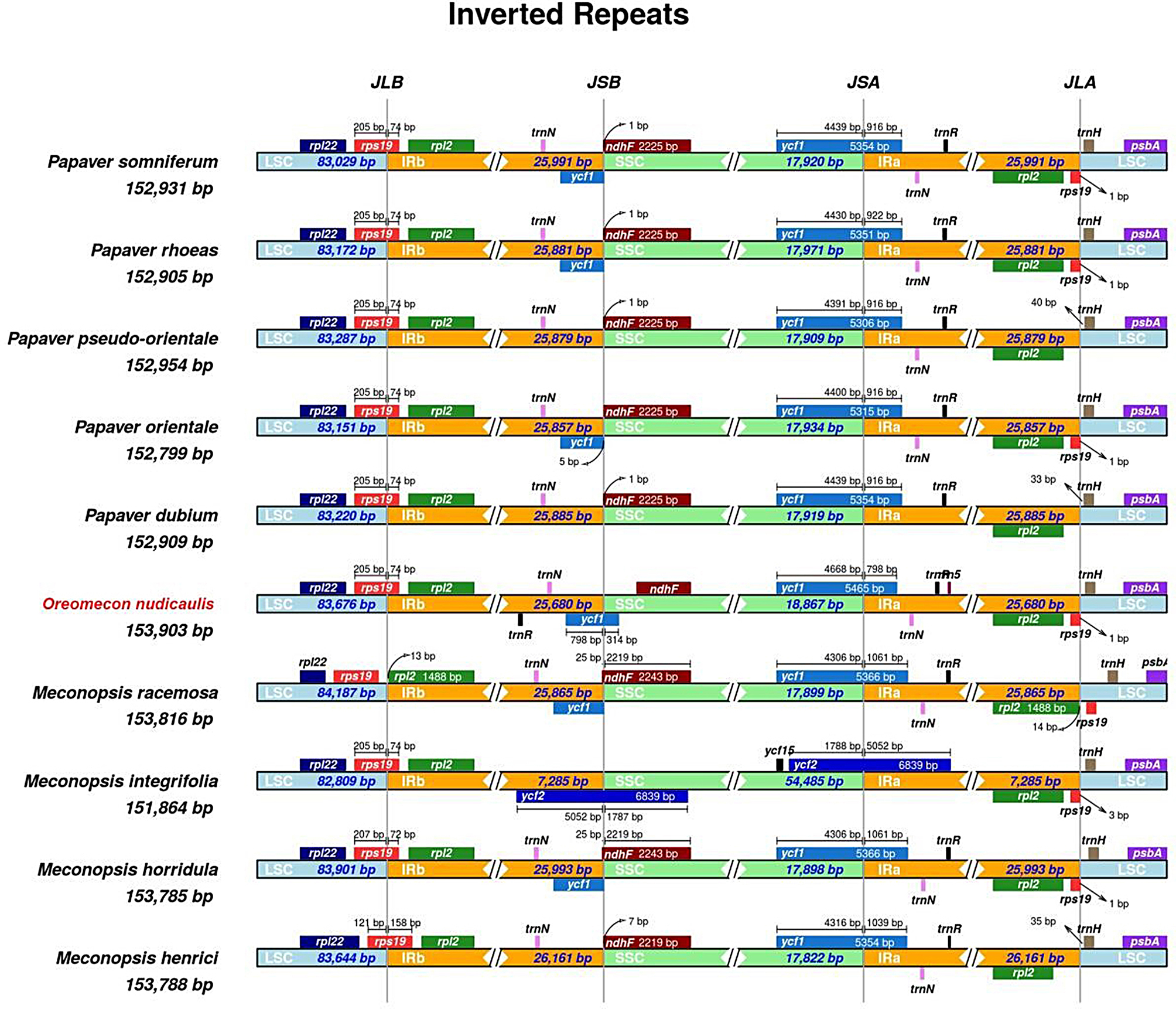
The comparison of Small Single-Copy (SSC), Large Single-Copy (LSC), Inverted Repeat B (IRB), and IRB boundary regions in the chloroplast genomes of *Meconopsis henrici* (MN488591), *Meconopsis horridula* (MK533646), *Meconopsis integrifolia* (MK533647), *Meconopsis racemosa* (MK533649), *O. nudicaulis* (MW151698), *Papaver dubium* (NC_065205), *Papaver orientale* (NC_037832), *Papaver pseudo-orientale* (NC_065210), *Papaver rhoeas* (NC_037831), *Papaver somniferum* (NC_029434) was conducted. Comparative analysis of junction sites in chloroplast genomes was also performed. The coordinate position of the start or end of each gene from the corresponding junction is shown with arrows. All the genes that integrate from one region of the chloroplast genome to another region are shown with the T bar above or below, indicating the length of base pairs for which the integration of genes has occurred. The junction sites between each corresponding two regions on the genome are denoted as JLA (IRa/LSC), JLB (IRb/LSC), JSA (SSC/IRa), and JSB (IRb/SSC).

The chloroplast genomes of the ten studied species, including five *Papaver* species (*P. somniferum*, *P. rhoeas*, *P. pseudo-orientale*, *P. orientale*, and *P. dubium*), one *Oreomecon* species (*O. nudicaulis*), and four *Meconopsis* species (*M. racemosa*, *M. integrifolia*, *M. horridula*, and *M. henrici*), exhibit the typical quadripartite structure consisting of a large single copy (LSC) region, a small single copy (SSC) region, and two inverted repeats (IRa and IRb) (Fig. 5). The genome sizes of the *Papaver* species range from 152,799 bp in *P. orientale* to 152,954 bp in *P. pseudo-orientale*, with the LSC region spanning from 83,029 bp to 83,287 bp and the SSC region ranging from 17,909 bp to 17,971 bp. The IRa and IRb regions in *Papaver* species are highly similar in size, ranging from 25,857 bp to 25,991 bp. The chloroplast genome of *O. nudicaulis* is slightly larger than those of *Papaver* species, with a total length of 153,903 bp, an LSC region of 83,676 bp, an SSC region of 18,867 bp, and IR regions of 25,680 bp each. Among the *Meconopsis* species, *M. integrifolia* has the smallest chloroplast genome (151,864 bp) and the most divergent structure, with significantly reduced IR regions (7,285 bp each) and an expanded SSC region (54,485 bp). The other three *Meconopsis* species have genome sizes ranging from 153,785 bp to 153,816 bp, with LSC regions spanning from 83,644 bp to 84,187 bp, SSC regions ranging from 17,898 bp to 17,899 bp, and IR regions ranging from 25,865 bp to 26,161 bp.

The gene content and order are largely conserved across the studied species, with notable genes such as *trnN*, *trnR*, *trnH*, *rpl22*, *rps19*, *ndhF*, *ycf1*, and *psbA* present in the chloroplast genomes. However, variations in the positioning of these genes relative to the IR boundaries are observed. In *Papaver* species, the rps19 gene is located in the LSC region, while in *O. nudicaulis* and *Meconopsis* species, it is situated in the IRb region. Similarly, the ndhF gene is found in the SSC region in *Papaver* species but extends into the IRb region in *O. nudicaulis*. The IR boundaries also exhibit some variability among the studied species. In *Papaver* species, the LSC/IRb boundary lies within the *rps19* gene, whereas in *O. nudicaulis* and *Meconopsis* species (except for *M. integrifolia*), it is located between the rpl22 and rps19 genes. The SSC/IRa boundary is generally situated within the ycf1 gene, but its exact position varies slightly among the species. *M. integrifolia* displays the most distinct IR boundary arrangement, with the LSC/IRb boundary located within the rpl22 gene and the SSC/IRa boundary situated within the ndhF gene. These findings highlight the evolutionary dynamics of chloroplast genomes in *O. nudicaulis*, *Papaver* and *Meconopsis* species, with both conserved structures and lineage-specific variations. The reduced IR regions and expanded SSC region in *M. integrifolia* suggest a unique evolutionary trajectory within the genus. The similarities between *O. nudicaulis* and *Meconopsis* species in terms of IR boundary arrangement and gene positioning support the close phylogenetic relationship between these genera.

### Phylogenetic analysis of the chloroplast genome of *O. nudicaulis*

**Fig. 6:**
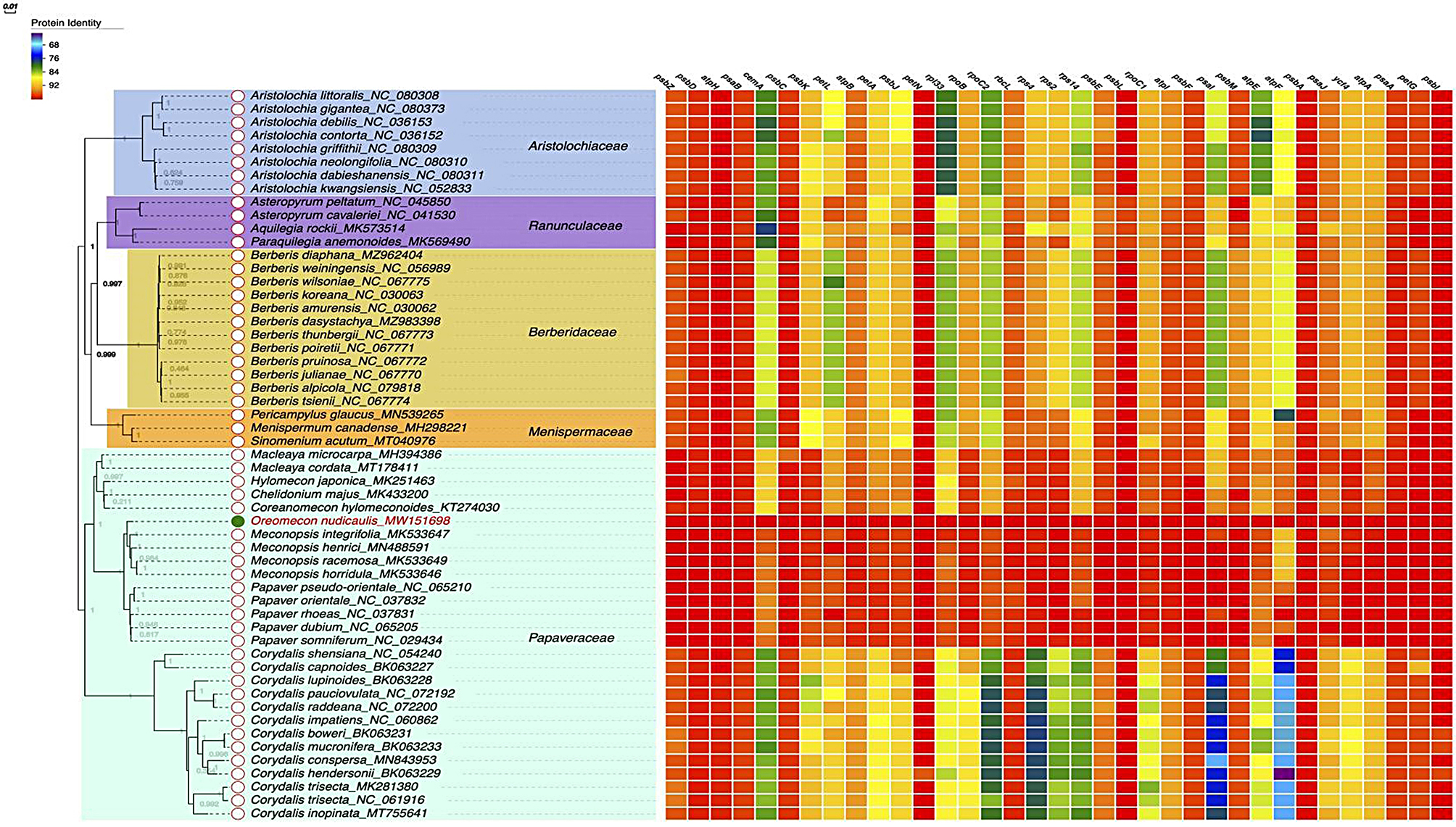
Phylogenetic tree constructed using the maximum likelihood (ML) method based on the complete chloroplast genome sequences of 55 Ranales species. The numbers at the nodes are the bootstrap support values. The heatmap adjacent to the phylogenetic tree displays the protein similarity of various species compared to *O. nudicaulis*. Protein identities range from 68–100%, indicating the level of similarity each species’ chloroplast genome shares with O. nudicaulis. The tree demonstrates the evolutionary relationships among the Aristolochiaceae, Ranunculaceae, Berberidaceae, Menispermaceae, and Papaveraceae families

The phylogenetic analysis of species from Aristolochiaceae, Ranunculaceae, Berberidaceae, Menispermaceae, and Papaveraceae families reveals a well-supported evolutionary history. The maximum likelihood tree, based on the comparative analysis of chloroplast genomes, provides insights into the relationships among these families and the species within them. The tree topology demonstrates a clear separation of the Aristolochiaceae family from the other four families, with *Aristolochia* species forming a monophyletic clade. This clade is supported by a bootstrap value of 1, indicating high confidence in the grouping. Within the *Aristolochia* clade, *A. littoralis* and *A. gigantea* are sister taxa, while *A. debilis* and *A. contorta* form another subgroup. *A. griffithii* is sister to a clade containing *A. neolongifolia*, *A. dabieshanensis*, and *A. kwangsiensis*, with varying levels of bootstrap support for the internal nodes. (Fig. 6). Ranunculaceae, represented by *Asteropyrum peltatum*, *A. cavaleriei, Aquilegia rockii*, and *Paraquilegia anemonoides*, is sister to a clade containing Berberidaceae, Menispermaceae, and Papaveraceae. Within Ranunculaceae, Asteropyrum species form a monophyletic group, while *Aquilegia rockii* and *Paraquilegia anemonoides* are sister taxa. The family is divided into two main subclades. The first subclade contains *Macleaya*, *Hylomecon*, *Chelidonium*, and *Coreanomecon* species, with *Macleaya microcarpa* and *M. cordata* forming a sister group. The second subclade comprises *Oreomecon*, *Meconopsis*, and *Papaver* species. The protein identity analysis, represented by the matrix on the right side of the figure, shows varying levels of similarity among the species for selected genes. For example, the psbZ gene exhibits high identity (100%) across all species, while other genes, such as atpH and psaB, show lower levels of identity, ranging from 21.1 to 100%. These identity values provide additional support for the phylogenetic relationships inferred from the tree.

The results indicated that the plants in the order Ranunculales clustered into two major clades, with Papaveraceae forming a distinct clade. Notably, our study focused on *O. nudicaulis*, which was originally classified under the genus *Papaver*, but the phylogenetic tree showed that it did not form a clade with other *Papaver* species. Instead, it clustered with the genus *Meconopsis*, and its closest relative was *Meconopsis racemosa*.

### Collinearity analysis of the chloroplast genome of *O. nudicaulis*

**Fig. 7:**
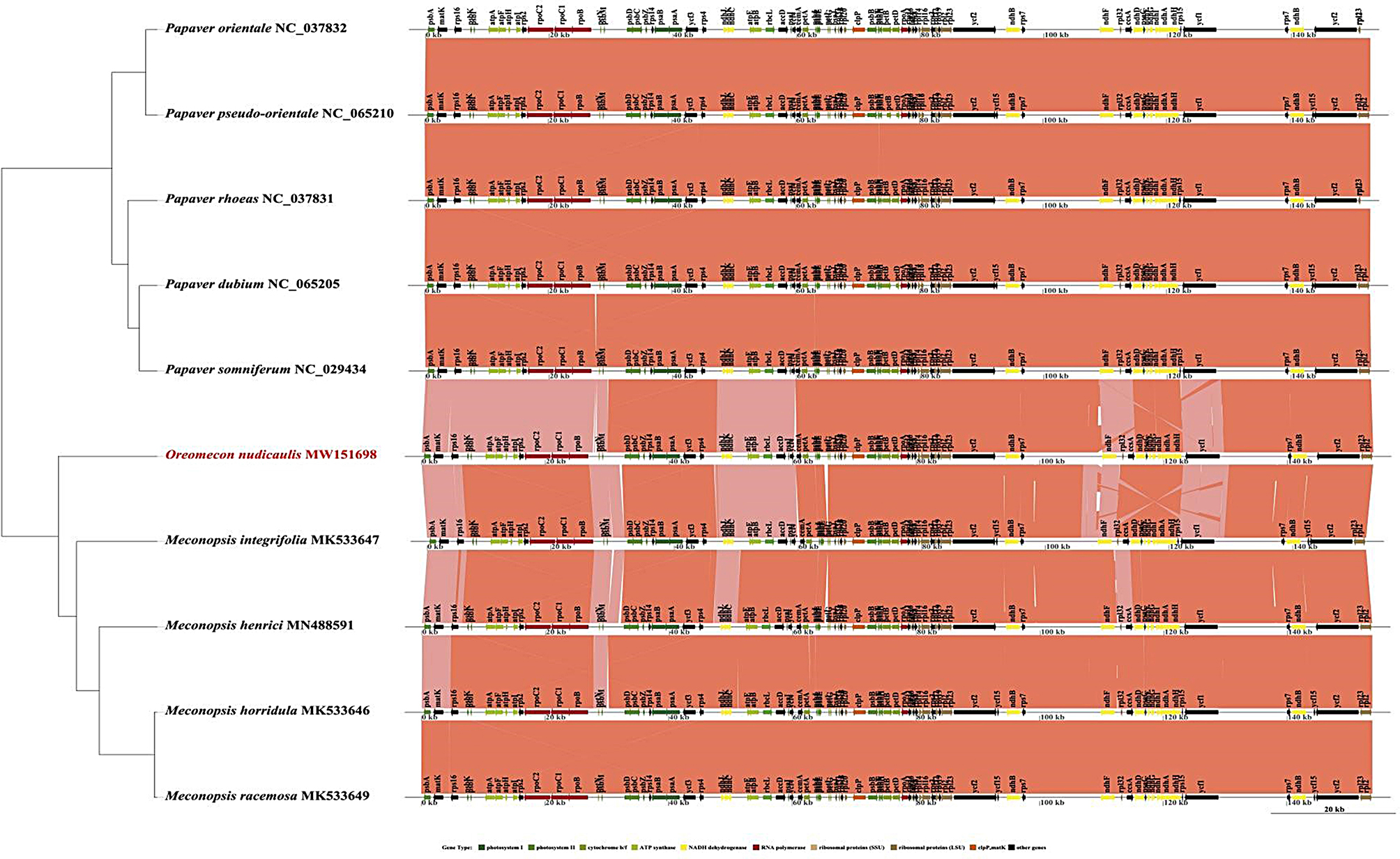
Collinearity analysis of chloroplast genomes related to *O. nudicaulis*. The figure compares the collinear blocks among the chloroplast genomes of O. nudicaulis and other related species including *Papaver orientale* (NC_037832), *Papaver pseudo-orientale* (NC_065210), *Papaver rhoeas* (NC_037831), *Papaver dubium* (NC_065205), *Papaver somniferum* (NC_029434), *Meconopsis integrifolia* (MK533647), *Meconopsis henrici* (MN488591), *Meconopsis horridula* (MK533646), and *Meconopsis racemosa* (MK533649). The x-axis represents the length of the chloroplast genome in kilobases (kb). Different colored blocks indicate conserved genomic regions, illustrating the synteny and genomic rearrangements among these species

The phylogenetic analysis presented in this scientific study utilizes a Maximum Likelihood Phylogenetic Tree to explore the evolutionary relationships among a select group of species from the Aristolochiaceae, Ranunculaceae, Berberidaceae, Menispermaceae, and Papaveraceae families. The tree is meticulously rooted to serve as a reference for tracing the evolutionary history, and it incorporates bootstrap values which are numerical indicators of the confidence in the branching patterns, with values nearing 1.0 reflecting strong support. In terms of the tree’s visualization, branch lengths are scaled to represent the number of substitutions per site, effectively serving as a metric for evolutionary distance. A scale bar is thoughtfully included, calibrated to indicate 0.01 substitutions per site, which aids in the interpretation of the branches’ lengths. Collinearity analysis revealed that the *Papaver* genome sequence exhibited high homology (Fig. 7). This homogeneity underscores the genetic stability and conservation within the genome. The chloroplast genomes of the three *Papaver* species connected with a line, indicating that the chloroplast genomes of these species were relatively conserved and that no rearrangement occurred in terms of gene organization. However, the vertical lines on the strip for the species *O. nudicaulis* represent its genes or genetic markers. These interconnected lines, which link the stripes of various species through vibrant hues, denote a striking similarity in the genes or markers located at these specific points. This resemblance strongly implies the existence of a shared ancestral origin or evolutionary conservation of these genetic elements across diverse species.

In a comparative genomic collinearity analysis conducted between the genera *Oreomecon* and *Papaver*, a suite of genes integral to photosynthetic functionality and gene expression—specifically *psbA*, *matK*, *rps16*, *psbK*, *atpA*, *atpF, atpH*, *atpI*, *rps2, rpoC2, rpoC1*, *rpoB*, *petN*, and *psbM*—demonstrated a moderate degree of conservation. This suggests that despite their crucial functions in photosynthesis and other pivotal biological processes, the sequences and structures of these genes have undergone significant evolutionary divergence across species belonging to these two genera. This divergence could reflect unique adaptive evolutionary trajectories, resulting in variations in the functional and regulatory mechanisms of these genes across different species.

## Discussion

The chloroplast genome of *O. nudicaulis*, a significant perennial herb recently reclassified from the genus *Papaver* to the genus *Oreomecon*, was sequenced, assembled, and annotated in this study. The results provide valuable insights into the genomic structure, gene content, and evolutionary relationships of *O. nudicaulis* within the Papaveraceae family. The chloroplast genome of *O. nudicaulis* exhibited a typical circular quadripartite structure, with a total length of 153,903 bp. The four regions included the LSC region (83,676 bp), SSC region (18,867 bp), and IRa/IRb region (25,680 bp each). The CG content in each region was lower than the AT content, with the SSC region showing the lowest CG content. A total of 130 genes and 2 pseudogenes (ycf1 and rps19) were annotated in the chloroplast genome of *O. nudicaulis*, with 18 genes containing introns, of which only 2 genes had 2 introns, while the rest had a single intron each. This gene content and structure are similar to those reported in other Papaveraceae species. Codon usage analysis revealed the presence of 65 codons, totaling 26,060, with the codon encoding leucine exhibiting the highest frequency (2,715 occurrences) and 32 codons identified with usage preferences. This finding aligns with observations in other angiosperm taxa, where leucine (Leu) is the most abundant amino acid, while cysteine (Cys) ranks as the least prevalent, excluding stop codons. Relative synonymous codon usage (RSCU) analysis revealed a bias in codon usage favoring A and U at the third codon position within the Papaveraceae family, corroborating findings from prior research.

Research on the phylogeny of the family Papaveraceae, particularly focusing on the genus *Papaver* L. and related genera, has undergone a series of relevant studies [32–34]. However, in the genus *Papaver*, almost all species exhibit similar flower shapes, colors, and fruits, making species identification based solely on morphological characteristics complicated [35, 36]. The establishment of the new genus *Oreomecon* in 2022 and the transfer of several species originally classified in the genus *Papaver* to this new genus have provided new insights into the taxonomy of the Papaveraceae family [9]. The phylogenetic analysis in the present study revealed that *O. nudicaulis* did not form a clade with other *Papaver* species, consistent with the revised taxonomy of the family Papaveraceae.

Comparative analysis of the IR boundary regions showed that the genomic structure of *O. nudicaulis* exhibits minimal variation compared to that of other plants in the same family, with relatively conserved gene types, positions, and boundary distances. This suggests a relatively low evolutionary rate among closely related species within the genus Oreomecon of the family Papaveraceae. Whether in terms of gene types, positions, or boundary distances, the structure remains relatively conserved. This suggests a relatively low evolutionary rate among closely related species within the genus *Oreomecon* of the family Papaveraceae. Codon usage patterns are fundamental genetic attributes of organisms and are intricately linked to mutations, natural selection, and a spectrum of other molecular evolutionary events [37]. Our investigations revealed that among all amino acids, leucine (Leu) was present at the highest frequency in *O. nudicaulis*. In contrast, cysteine (Cys) ranks as the least prevalent amino acid, excluding stop codons, a trend that aligns with observations in other angiosperm taxa [38]. Moreover, relative synonymous codon usage (RSCU) analysis revealed that codons tend to terminate in A or U when the RSCU value exceeds unity. Conversely, codons predominantly end in C or G when the RSCU value falls below one. This pattern underscores a bias in codon usage favoring A and U at the third codon position within the Papaveraceae family, corroborating findings from prior research [35, 36].

Microsatellites or simple sequence repeats (SSRs) are highly polymorphic repetitive DNA sequences with extensive applications as molecular markers in species identification, phylogenetic research, and population genetics. Due to their remarkable polymorphic nature, SSRs have extensive applications as molecular markers in tasks such as species identification, phylogenetic research, and population genetics [39, 40]. In this study, the chloroplast genome of *O. nudicaulis* encompassed a total of 129 microsatellite sequences, which is less than that of other *Papaver* species In a previous study, a total of 180–190 SSRs were identified in the chloroplast genomes of *Papaver* species [35]. However, the predominance of shorter repeat units in *O. nudicaulis* SSRs makes them particularly useful for developing effective molecular markers, as they tend to exhibit greater polymorphism. Thus, *O. nudicaulis* SSRs, which are predominantly composed of shorter units, hold great potential for developing effective molecular markers. These markers hold great potential for future population genetics and evolutionary studies, offering insights into the biology and history of *O. nudicaulis* and related species within the Papaveraceae family.

Previous studies on *O. nudicaulis* have largely focused on its biological characteristics, seed germination, chemical composition, introduction and cultivation, and population genetics [41]. However, there is currently no reported research on the genomics of *Oreomecon*. This study addresses this gap by conducting chloroplast genome sequencing and analysis of the genomic sequence and structural features of *O. nudicaulis*. The construction of a phylogenetic tree based on the genomic data provides insights into the systematic evolution and phylogenetic relationships of this species This research aimed to understand systematic evolution and phylogenetic relationships by constructing a phylogenetic tree. The findings of this study not only contribute to the genetic information on the chloroplast genome of the family Papaveraceae but also provide fundamental data for the classification status, systematic origin, species identification, and molecular marker development of wild poppy.

## Conclusion

This study presents the first comprehensive analysis of the chloroplast genome of *O. nudicaulis*, revealing its genomic structure, gene content, and evolutionary relationships within the Papaveraceae family. The findings support the recent taxonomic revision of the genus *Oreomecon* and highlight the significance of chloroplast genome data in resolving phylogenetic relationships. The identified repetitive elements and SSRs serve as valuable resources for future genetic studies and molecular marker development in *O. nudicaulis* and related species. The comparative analysis of IR boundaries and codon usage patterns contributes to our understanding of the evolutionary dynamics and conservation of chloroplast genomes within the Papaveraceae family. This study lays a foundation for further research on the genetic diversity, evolutionary history, and potential applications of *O. nudicaulis* in traditional medicine and horticulture. Future studies focusing on the functional and regulatory mechanisms of genes and comparative analysis across a wider range of Papaveraceae species will provide additional insights into the evolutionary adaptations and diversification within this family.

### Electronic supplementary material

Below is the link to the electronic supplementary material.

Supplementary Material 1

## Data Availability

The chloroplast genome of O. nudicaulis is available at GenBank under accession number MW151698. The raw sequencing data have been deposited in the Sequence Read Archive (SRA) of NCBI under accession number SRR27751561. The Bioproject_accession number is PRJNA1068053. The accession number in this study include Aristolochia littoralis NC_080308, Aristolochia gigantea NC_080373, Aristolochia debilis NC_036153, Aristolochia contorta NC_036152, Aristolochia griffithii NC_080309, Aristolochia neolongifolia NC_080310, Aristolochia dabieshanensis NC_080311, Aristolochia kwangsiensis NC_052833, Asteropyrum peltatum NC_045850, Asteropyrum cavaleriei NC_041530, Aquilegia rockii MK573514, Paraquilegia anemonoides MK569490, Berberis diaphana MZ962404, Berberis weiningensis NC_056989, Berberis wilsoniae NC_067775, Berberis koreana NC_030063, Berberis amurensis NC_030062, Berberis dasystachya MZ983398, Berberis thunbergii NC_067773, Berberis poiretii NC_067771, Berberis pruinosa NC_067772, Berberis julianae NC_067770, Berberis alpicola NC_079818, Berberis tsienii NC_067774, Pericampylus glaucus MN539265, Menispermum canadense MH298221, Sinomenium acutum MT040976, Macleaya microcarpa MH394386, Macleaya cordata MT178411, Hylomecon japonica MK251463, Chelidonium majus MK433200, Coreanomecon hylomeconoides KT274030, Meconopsis integrifolia MK533647, Meconopsis henrici MN488591, Meconopsis racemosa MK533649, Meconopsis horridula MK533646, Papaver pseudo-orientale NC_065210, Papaver orientale NC_037832, Papaver rhoeas NC_037831, Papaver dubium NC_065205, Papaver somniferum NC_029434, Corydalis shensiana NC_054240, Corydalis capnoides BK063227, Corydalis lupinoides BK063228, Corydalis pauciovulata NC_072192, Corydalis raddeana NC_072200, Corydalis impatiens NC_060862, Corydalis boweri BK063231, Corydalis mucronifera BK063233, Corydalis conspersa MN843953, Corydalis hendersonii BK063229, Corydalis trisecta MK281380, Corydalis trisecta NC_061916, Corydalis inopinata MT755641.
