# Supplementary material for: Comparative chloroplast genomics and phylogenetic analysis of Oreomecon nudicaulis (Papaveraceae)
Source: BMC Genom Data. 2024 May 30;25:49. doi: 10.1186/s12863-024-01236-8 (PMC11141030; doi:10.1186/s12863-024-01236-8)
Supplement: Supplementary file 1 — Supplementary Material 1 [file 12863_2024_1236_MOESM1_ESM.docx]

**Table 1s** Codons usage frequency of the complete chloroplast genome of *O. nudicaulis*

| Symbol | Codons | Number | RSCU | Symbol | Codons | Number | RSCU |
| --- | --- | --- | --- | --- | --- | --- | --- |
| GCA | Ala | 373 | 1.093 | CUU | Leu | 605 | 1.3434 |
| GCC | Ala | 236 | 0.6916 | UUA | Leu | 788 | 1.7498 |
| GCG | Ala | 154 | 0.4513 | UUG | Leu | 553 | 1.228 |
| GCU | Ala | 602 | 1.7641 | AAA | Lys | 970 | 1.4554 |
| AGA | Arg | 471 | 1.8446 | AAG | Lys | 363 | 0.5446 |
| AGG | Arg | 172 | 0.6736 | AUG | Met | 626 | 1 |
| CGA | Arg | 328 | 1.2846 | UUC | Phe | 545 | 0.7435 |
| CGC | Arg | 103 | 0.4034 | UUU | Phe | 921 | 1.2565 |
| CGG | Arg | 123 | 0.4817 | CCA | Pro | 313 | 1.155 |
| CGU | Arg | 335 | 1.312 | CCC | Pro | 201 | 0.7417 |
| AAC | Asn | 295 | 0.486 | CCG | Pro | 146 | 0.5387 |
| AAU | Asn | 919 | 1.514 | CCU | Pro | 424 | 1.5646 |
| GAC | Asp | 246 | 0.4526 | AGC | Ser | 144 | 0.4192 |
| GAU | Asp | 841 | 1.5474 | AGU | Ser | 402 | 1.1703 |
| UGC | Cys | 99 | 0.6226 | UCA | Ser | 431 | 1.2547 |
| UGU | Cys | 219 | 1.3774 | UCC | Ser | 348 | 1.0131 |
| CAA | Gln | 677 | 1.5128 | UCG | Ser | 183 | 0.5328 |
| CAG | Gln | 218 | 0.4872 | UCU | Ser | 553 | 1.6099 |
| GAA | Glu | 964 | 1.4367 | UAA | Ter* | 47 | 1.5326 |
| GAG | Glu | 378 | 0.5633 | UAG | Ter* | 24 | 0.7826 |
| GGA | Gly | 677 | 1.5291 | UGA | Ter* | 21 | 0.6848 |
| GGC | Gly | 174 | 0.393 | ACA | Thr | 405 | 1.2263 |
| GGG | Gly | 327 | 0.7386 | ACC | Thr | 251 | 0.76 |
| GGU | Gly | 593 | 1.3394 | ACG | Thr | 167 | 0.5057 |
| CAC | HIS | 156 | 0.4867 | ACU | Thr | 498 | 1.5079 |
| CAU | HIS | 485 | 1.5133 | UGG | Trp | 467 | 1 |
| AUA | Ile | 661 | 0.9332 | UAC | Tyr | 179 | 0.3776 |
| AUC | Ile | 447 | 0.6311 | UAU | Tyr | 769 | 1.6224 |
| AUU | Ile | 1017 | 1.4358 | GUA | Val | 517 | 1.4512 |
| CUA | Leu | 374 | 0.8305 | GUC | Val | 184 | 0.5165 |
| CUC | Leu | 195 | 0.433 | GUG | Val | 197 | 0.553 |
| CUG | Leu | 187 | 0.4152 | GUU | Val | 527 | 1.4793 |
| GCA | Ala | 373 | 1.093 | CUU | Leu | 605 | 1.3434 |

Note: “*” indicates that the gene is a termination codon.

Note: “*” indicates that the gene is a termination codon.
